# Co-variation of *STI1* and *WDR36/UTP21* alters cell proliferation in a glaucoma model

**Published:** 2011-07-19

**Authors:** Tim Footz, Stéphane Dubois, Mansoor Sarfarazi, Vincent Raymond, Michael A. Walter

**Affiliations:** 1 Department of Medical Genetics, University of Alberta, Edmonton, Alberta, Canada; 2 Department of Molecular Medicine, Université Laval, Québec City, QC, Canada; 3 Molecular Ophthalmic Genetics Laboratory, University of Connecticut Health Center, Farmington, CT

## Abstract

**Purpose:**

To investigate the role of multigenic variation in primary open-angle glaucoma (POAG) involving the rRNA processing gene WD repeat domain 36 (*WDR36*).

**Methods:**

We examined the heat shock protein 70/90 (HSP70/90)-organizing co-chaperone stress-induced-phosphoprotein 1 (*STI1*) as a potential co-modifying gene in glaucoma patients found to harbor WDR36 amino acid variation. The *STI1* gene was sequenced and its POAG-associated amino acid variant K434R, as well as the single nucleotide polymorphism (SNP) P173T, were tested for functional defects in a yeast model system previously used to characterize WDR36 variants (using the homologous yeast gene U3 protein 21 [*UTP21*]).

**Results:**

A POAG patient heterozygous for the WDR36 variant L25P was discovered to also carry the STI1 variant K434R in a heterozygous state. Variant K434R, located at an evolutionarily-conserved site, was not found in a pool of clinically-examined individuals lacking *WDR36* variation which included 55 normal controls and 20 patients with normal tension glaucoma (NTG). STI1 (K434R) and the homologous yeast variant K470R were able to rescue yeast growth-inhibition by the HSP90-inhibitor radicicol. Double mutant haploid strains expressing human STI1 (K434R) and recombinant yeast UTP21 variants did not have significantly different levels of 18S rRNA from the corresponding hSTI1 (WT) strains. However, specific double mutant K434R strains exhibited significantly slower culture growth at 37 °C. Double mutant P173T strains also displayed altered growth rates at 37 °C.

**Conclusions:**

*STI1* variation does not play a significant direct role in the genetics of POAG. However, as previously found for the *STI1* null allele, non-synonymous variants of human *STI1* confer growth dysregulation in the context of specific yeast *UTP21* mutations and heat stress. Based on the genetic association of two co-heterozygous *STI1* and *WDR36* variants in a POAG patient and the functional analyses performed in a model system for basic eukaryotic cellular processes, these experiments point to a conserved molecular pathway involving *STI1* and *WDR36*.

## Introduction

Glaucoma, a leading cause of blindness worldwide [1], is characterized by optic nerve atrophy following progressive loss of retinal ganglion cells (RGCs). Primary open angle glaucoma (POAG, OMIM 137760), without a known secondary cause such as anatomic impairment of the aqueous humor outflow pathway, is the common form of adult-onset glaucoma, for which elevated intraocular pressure (IOP) is a risk factor [2]. High IOP may result nonetheless from defective aqueous humor outflow causing stress-induced apoptosis of RGCs at the optic nerve head [3-5]. Family history of the disease is also a major risk factor of glaucoma indicating there is a strong genetic component to its etiology [2]. Numerous chromosomal loci have been mapped for involvement in familial forms of POAG [6,7]. However, only five causative genes have been identified through linkage mapping and genome-wide association studies, accounting for less than ten percent of the heritability of POAG. These five genes are myocilin (*MYOC*/GLC1A; OMIM 601652) [8], optineurin (*OPTN*/GLC1E; OMIM 602432) [9], WD repeat domain 36 (*WDR36*/GLC1G; OMIM 609669) [10], cytochrome P450, family 1, subfamily B, polypeptide 1 (*CYP1B1*) (OMIM 601771) [11] and neurotrophin 4 (*NTF4*) (OMIM 162662) [12].

Multigenic inheritance and environmental interactions contribute to the complex etiology of POAG [13,14]. We previously discovered that variants in yeast U3 protein 21 (*UTP21*), homologous to those found in *WDR36* in glaucoma patients, alter cell proliferation in strains deficient for the co-chaperone protein stress-induced-phosphoprotein 1 (Sti1p) [15]. The nature of the biochemical interaction of these two genes in yeast is unclear. Utp21p is a core member of the small subunit (SSU) processome, a protein complex responsible for the initial cleavage steps that process the 35S rRNA transcript into 18S rRNA, a structural component of ribosomes [16] and functionally homologous roles have been established for zebrafish [17] and mouse [18] WDR36. Sti1p is an adaptor protein in the heat shock protein 70/90 (HSP70/90) chaperone apparatus [19]. It is not known if any rRNA processing proteins are bona fide clients of the HSP70/90 chaperone system, nor if Utp21p has a novel role in Sti1p pathways. However, it may be possible that the additive effects of perturbations in two separate gene networks can overwhelm cell homeostasis or responses to stress.

Sti1p (also called HOP and STIP1 in mammals) was identified as a stress-inducible protein in yeast required for survival at low and high temperatures [20]. The transfer of incorrectly-folded client proteins between the eukaryotic ATP-dependent chaperones HSP70 and HSP90 is mediated by interactions with STI1 along with other co-chaperones [19,21]. The majority of these client proteins, such as steroid hormone receptors, kinases and transcription factors, are involved in signal transduction and are activated by conformational changes arising from interaction with the chaperone machinery [22]. The structure of STI1 is characterized by three helix-rich tetratricopeptide (TPR) domains: TPR1 at the NH_2_-terminus is important for binding HSP70, TPR2A, located centrally, is required for binding HSP90 and STI1 dimerization, and the COOH-terminal TPR2B domain contributes to in vivo interactions involving the above components [23-26]. In addition to being well conserved at the sequence level among eukaryotes, human STI1 functionally complements the lack of yeast Sti1p in HSP70/90-mediated assembly of the glucocorticoid receptor [24].

Recently, novel roles for mammalian STI1 as a neuroprotective cell-surface ligand for cellular prion protein (PrP^c^) have emerged. The interaction of STI1 and PrP^c^ promotes neuritogenesis and cell survival in hippocampal neurons [27]. STI1 reduces apoptotic cell death in the neuroblastic layer of retinal explants only in mice that express PrP^c^ [28]. However, STI1 can also partake in PrP^c^-independent modulation of cell proliferation and survival in the developing retina [29]. These findings indicate that STI1 may participate in an extracellular chaperone system to transmit neurotrophic effects on damaged retinal tissue and thus could be important to glaucoma pathogenesis. Additionally, the neurotrophic interaction of STI1 and PrP^c^ has been shown to stimulate neuronal protein synthesis [30], which may have implications specifically for POAG patients defective in the ribosome biosynthesis gene *WDR36*.

In this study we investigated whether the genetic interaction of *UTP21* with *STI1* was conserved in humans and could help to explain the ambiguity of associating isolated *WDR36* variants with POAG (reviewed in [4]). POAG patients heterozygous for *WDR36* non-synonymous variants were sequenced for coding and splice-site alterations in *STI1*. One non-synonymous *STI1* variant was discovered which alters an evolutionarily-conserved residue in the TPR2B domain. Using expression analyses in haploid yeast, our findings reveal that the K434R variant does not profoundly affect HSP90 function or rRNA processing, but does confer a proliferative disadvantage in specific *UTP21* mutant backgrounds. Additionally, experiments with a second non-synonymous variant in a conserved residue, P173T, which was previously deposited into the NCBI dbSNP Short Genetic Variation database, confirm that mutant forms of Sti1p and Utp21p interact to regulate cell proliferation in response to heat stress.

## Methods

### Recruitment and clinical assessment

This research was approved by the CHUL Research Center Ethics Committee (Québec, QC) and followed the tenets of the Declaration of Helsinki. Recruitment was performed through ophthalmologists in the Province of Québec, Canada, and New England, USA. Only unrelated Caucasian individuals were studied and, all participants, affected or not, signed an informed consent document before entering the study. In families affected by glaucoma, only one patient was asked to enter the study.

Clinical assessment comprised complete ophthalmologic evaluation as described in our earlier study [31]. Diagnostic criteria for POAG were: (i) characteristic optic disc damage and/or visual ﬁeld impairment, (ii) grade III/IV (open-angle) gonioscopy and (iii) IOPs above 21 mmHg in at least one eye. Persons were considered normal when they presented normal optic discs and showed highest IOPs ever recorded at <22 mmHg. Subjects were diagnosed with normal-tension glaucoma (NTG) when they presented optic disc damage, visual ﬁeld impairment and open angles with IOP <22 mmHg.

### *WDR36* screening

Patient genomic DNA was extracted from peripheral blood using the Puregene DNA isolation protocol (Sigma-Aldrich, Oakville, ON). Each of the 23 *WDR36* coding exons and flanking introns was screened by direct genomic sequencing in a minimum of 284 POAG patients with ocular hypertension and 31 normal tension glaucoma (NTG) subjects. Fifty-six investigated asymptomatic subjects served as controls for all exons. WDR36 amplicons were obtained by Polymerase Chain Reaction (PCR) in a total volume of 50 μl using 70 ng of genomic DNA, 10 pmol of each primer, 200 μM dNTPs, 50 mM KCl, 20 mM Tris-HCl (pH 8.4), 1.5 mM MgCl_2_, and 1 U JumpStart™ Taq polymerase (Sigma-Aldrich). They were amplified following these conditions: a ‘hot-start’ of 5 min at 95 °C; 35 cycles of denaturation (94 °C for 30 s), annealing (58 °C for 30 s) and elongation (72 °C for 30–90 s); followed by one last elongation period of 7 min. For difficult amplicons, specific parameters were modified: lower annealing temperature, longer elongation time, use of betaine (1.2 M final concentration), or higher MgCl_2_ concentration. Primers used are listed in Table 1. *WDR36* amplicons were then purified on a fiberglass membrane (UNIFILTER®; Whatman, Piscataway, NJ). PCR incorporating the sequencing dye (BigDye®; Applied Biosystems, Carlsbad, CA) used a protocol consisting of an initial denaturation step of 30 s at 96 °C followed by 25 cycles of denaturation (96 °C for 10 s), annealing (53 °C for 5 s) and elongation (59 °C for 3 min). PCR products were purified by the Applied Biosystems ethanol-EDTA precipitation protocol and resuspended in a 50% Hi-Di™ Formamide solution. Samples were then resolved on the Applied Biosystems 3730xl platform. Sequence data were analyzed using the Staden preGap4 and Gap4 programs [32].

**Table 1:** Primers used in this study for screening *WDR36* and *STI1*.

**Amplicon**	**Forward PCR primer**	**Reverse PCR primer**
***WDR36***		
Exon 1	ttctgtcggaacctaacgagc	gagttagaggccaaggaggg
Exon 2	agtaagtgtctttcttatgaagg	atacatgttaccttggcttcc
Exon 3	ggacaaggtgatttcctatcc	ttctgaaaatttatcttcctccag
Exon 4	gagcagatgaacatgcctgg	ctctggcatagatgtttacatag
Exon 5	tagattagtatctaagtctgtgg	tgttatttatagacaaccctcca
Exon 6	atcagaatagtgtgtggaagag	aacactatttcctcaggctaac
Exon 7–9^@^	cttgaatggtaatacttgctgag	ggactcttagttttacccagac
Exon 10	tttaatgaagtagttgcaatctgg	cttatagcagaacctcaatcac
Exon 11	agtggtaataacatctttgttttg	acaagaccagcatgcacctg
Exon 12	cttgttagattggaaacatattgc	aatattatgatgagaaaccttgag
Exon 13–15	tgaaggctagtcccatatatag	atttgactgcatcaactccctg
Exon 16	taaggcagcctgaatgttagtc	gtttcatgacactactacctgc
Exon 17	gtggtgactttctgatcaatgc	agctgtctacattatcaagcag
Exon 18,19	gatggcatctatgacataagtc	gcattgtcagtgctgtcttac
Exon 20,21	ctgtggtattggtcagaagag	atcttaactactgagaacgctg
Exon 22,23	ttggtgtcatcgtttgtactg	ggaggtgcgattttatttcaag
***STI1***		
Exon 1	tgcaaaagaccaatccgtgtc	tcacccaagctgtggcctc
Exon 2	tggctccttagggaaggtgtga	actatcagtgtatcaggacagg
Exons 3,4	ctctttggccttcatgtgaatac	accctctgctgtgaggttacag
Exon 5	atgagtggtgcaaaatctagcca	ggtcaatcaaggtcaacagatg
Exons 6–8^*^	gccttcaggagaacttgatag	taaaacagtaagaaacaatggctc
Exons 9,10	cctaatgtgatttttaagcccaa	gtgagacactgtctcaaacatc
Exons 11–14^#^	tgggtgaggcattagagttgga	agagagatgaggccttctctc

@Additional sequencing primer: aatagaagtgaatgaatactggag.

*Additional sequencing primer: gccatagaatctgctgtgttaaga.

#Additional sequencing primer: cctgtcacctccatttctctc.

### *STI1* screening

Mutation analysis of the *STI1* gene was performed on a minimum of 118 POAG patients in which *WDR36* non-synonymous amino-acid variations had been previously detected (see above), as well as on a minimum of 20 NTG subjects and 55 asymptomatic controls that were determined to be wild-type for *WDR36*. The coding sequence and splice site junctions of *STI1* were sequenced in 13 amplicons using the same procedures as those described above for *WDR36*, except that different primers were used, listed in Table 1. All variations were confirmed by sequencing both strands of the PCR products.

### Sequence Alignment

**Figure 1:**
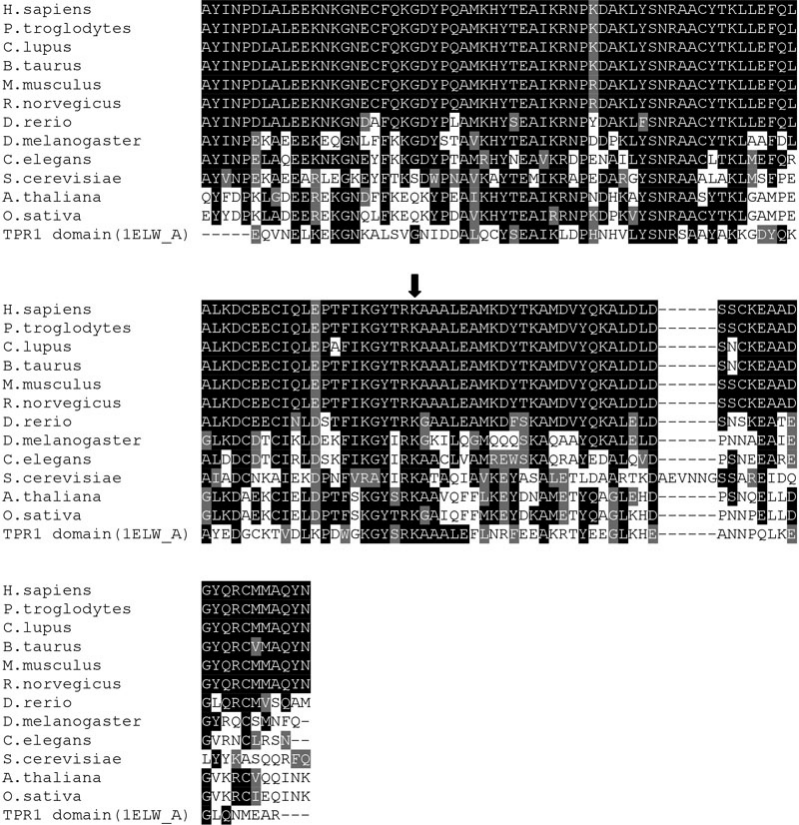
Alignment of TPR2B domains of STI1. Amino acids 353 to 477 of human STI1 (*H. sapiens*, GenBank NP_006810) are shown aligned to animal, fungal and plant STI1 sequences demonstrating evolutionary conservation of the TPR2B domain and residue K434 (indicated by the arrow). Alignment to the human TPR1 domain was used for homology modeling (see Figure 2). Black and gray shading represent identical and functionally-similar residues, respectively.

ClustalW2 was used to generate the alignment of the TPR2B domains of STI1 from sequences downloaded using Homologene: *Homo sapiens* (NP_006810), *Pan troglodytes* (XP_508521), *Canis familiaris* (XP_854960), *Bos taurus* (NP_001030569), *Mus musculus* (NP_058017), *Rattus norvegicus* (NP_620266), *Danio rerio* (NP_001007767), *Drosophila melanogaster* (NP_477354), *Caenorhabditis elegans* (NP_503322), *Saccharomyces cerevisiae* (NP_014670), *Arabidopsis thaliana* (NP_172691), and *Oryza sativa* (NP_001047563). The Boxshade server was used to prepare the figure (Figure 1).

### Homology modeling

The ClustalW2 alignment of the TPR2B domains was uploaded to HHpred where the TPR1 domain of STI1/HOP (PDB# 1elw_A) was identified as the highest-scoring structural homolog hit (*E*-value=1.3E-26). The TPR2B-1elw_A alignment was exported to Modeller within the same web platform to automatically generate the homology model that was saved and rendered using Swiss-PdbViewer [33] and POV-Ray. In silico mutagenesis was performed with Swiss-PdbViewer and model evaluations were performed with the ANOLEA server.

### Plasmid constructs

The plasmid pRS317:y*STI1* [34] (endogenous yeast *STI1* promoter and coding sequence) was a gift from Dr. Jill Johnson (Department of Microbiology, Molecular Biology and Biochemistry, University of Idaho, Moscow, ID). A human *STI1* cDNA (clone #2823157) was purchased from Open Biosystems (Huntsville, AL). An EcoRI/PstI fragment of the y*STI1* ORF was subcloned into vector pOTB7 which served as template for in vitro mutagenesis using the QuikChange® Site-Directed Mutagenesis Kit (Stratagene, La Jolla, CA) using the K470R primers (5′-GAG CTT ATA TCA GAA GGG CCA CCG CAC AAA TTG-3′, plus the reverse complement), then recombined back into the original pRS317:y*STI1*. The yeast *STI1* promoter (forward, 5′-CTG CAG GAA TTG ACC AAA CTA TTG AAC-3′; reverse, 5′-GAA TTC CTT TCT TGC GTA TAG GTT GTT-3′) and human *STI1* ORF (forward, 5′-GAA TTC ATG GAG CAG GTC AAT GAG CTG-3′; reverse, 5′-GAA TTC TCA CCG AAT TGC AAT CAG ACC-3′) were PCR-amplified and cloned separately into vector pGEM-T then recombined into pRS317 using PstI and EcoRI sites. The h*STI1* pGEM-T clone also served as template for mutagenesis with the K434R primers (5′-AGG GTT ATA CAC GGA GAG CCG CTG CGC TGG A-3′, plus the reverse complement) or P173T primers (5′-CTG GGC ACG AAA CTA CAA GAT ACC CGG ATC ATG-3′, plus the reverse complement). The mutagenized EcoRI fragment was subcloned into pRS317:y*STI1*prom-h*STI1* to replace the wild-type ORF. All vector inserts were verified by direct sequencing.

### Yeast assays

*Saccharomyces cerevisiae* strain CN11 (*sti1::HIS3*) was a gift from Dr. Jill Johnson (Department of Microbiology, Molecular Biology and Biochemistry, University of Idaho, Moscow, ID). Double mutant (*sti1/utp21Δ*) strains and recombinant *UTP21* constructs were previously described [15].

Yeast were cultured in YPD (10 g/l yeast extract, 20 g/l peptone, 2% D-glucose) or Synthetic drop-out (6 g/l nitrogen base, 2% D-glucose) media at 30 °C or 37 °C. Strain and plasmid identification was confirmed by standard PCR analysis of colony pellets by dispersing cells into 100 µl of 50 U/ml lyticase (#L4025; Sigma-Aldrich) in TE buffer, incubating at 37 °C for 30 min, then using 1 µl as template. Yeast transformation was performed by following a standard lithium acetate procedure followed by selection on appropriate dropout media.

Radicicol (Cayman Chemical, Ann Arbor, MI) was dissolved in DMSO (1 mg/0.5 ml) and added to cooling YPD+agar media to a final concentration of 39 µM for solid-support plates. Overnight cultures of yeast were diluted with water to an optical density (at 600nm) of OD_600_=0.5, then serially-diluted 10-fold, four times. Five microlitres of each dilution were spotted onto YPD+radicicol or YPD+DMSO plates and incubated at 30 °C for 48 h.

Total RNA was prepared from yeast cultured in complete media using a hot-phenol protocol [35]. Approximately 250 ng of RNA was subjected to electrophoresis in RNA Nano Chips in an Agilent 2100 Bioanalyzer (Agilent Technologies, Mississauga, ON). Triplicate samples were analyzed for the ratio of 18S/25S rRNA peak areas. Statistical significance of deviation (p<0.05) was calculated by two-tailed unequal-variance Student’s *t*-Tests in Microsoft Excel (Microsoft Canada Co., Mississauga, ON).

To measure growth curves, yeast were cultured in selective media at 30 °C overnight, with shaking at 225 rpm. Cultures were diluted in complete YPD media to reach an OD_600_ of ~0.20 in ~20 ml and then incubated at 37 °C. Aliquots (1 ml) were extracted every 2 h to monitor the OD_600_ as a function of cell concentration. Readings from four experiments were averaged and plotted with error bars representing standard error of the mean. Statistical significance of deviation (p<0.05) was calculated for linear trendlines by *t*-Tests in Microsoft Excel (Microsoft Canada Co.).

## Results

### Patient sequencing

To assess if *STI1* may be a co-modifier gene interacting with *WDR36* to affect the severity of the glaucoma phenotype, we screened for variations in each exon and flanking introns of the *STI1* gene in a minimum of 118 POAG patients carrying *WDR36* variations (data not shown), and in 75 individuals with no *WDR36* variation (20 NTG patients and 55 normal controls); additional information from partially-tested samples was included where the data were of sufficient quality to analyze. Overall, six heterozygous *STI1* sequence alterations were detected in 33 subjects (Table 2). Four of these encoded synonymous amino acid changes: R142R, T243T, Y248Y, and A437A. One variation was in intron 11, ivs11+10 T>G but is not predicted to change the splicing pattern of the *STI1* transcript. A single non-synonymous variant, K434R, was found in a heterozygous state in a heterozygous *WDR36*^+/L25P^ background, representing a potential genetic modification of the glaucoma phenotype in this individual. This male patient was diagnosed with POAG at age 43 years old. Although intraocular pressures in both eyes were not highly elevated at 22 mmHg at that age, his right and left visual fields presented features typical of moderate POAG and both optic nerve heads displayed advanced atrophy with cup-to-disk ratios estimated at 0.9 out of 1 (data not shown). Segregation studies of each variation could not be performed in this patient’s family. Eleven other POAG patients were also heterozygous for *WDR36*^L25P^. However, none of them were found to carry an *STI1* variation. The median of their ages-at-diagnosis was 59 years and ranged from 41 to 85 years old; the patient diagnosed at 41 being the only one diagnosed earlier than our *WDR36*^+/L25P^; *STI1*^+/K434R^ patient.

**Table 2:** Frequencies of heterozygous STI1 Variants discovered in this study.

***STI1* Variant**	***WDR36*-heterozygous POAG patients**	***WDR36*-wildtype NTG patients**	**Normal control individuals**
R142R	0/131	0/21	0/55
T243T	0/135	0/21	1/55
Y248Y	1/135	0/21	0/55
ivs11+10 T>G	1/118	0/20	0/56
K434R	1/124	0/21	0/56
A437A	14/124	3/21	12/56

### In silico analyses of the K434R variant

**Figure 2:**
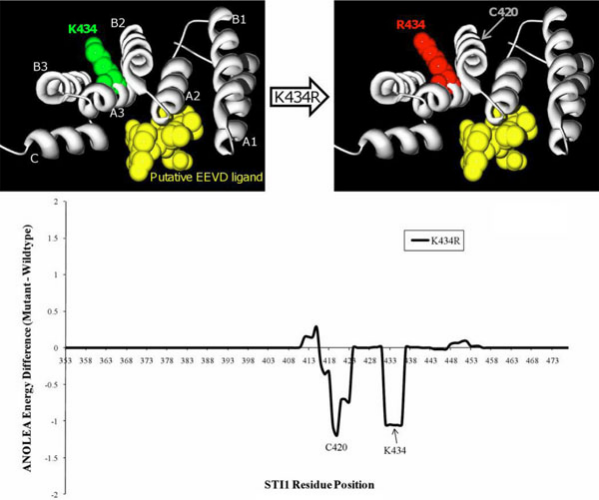
Homology modelling of K434R. A model of STI1's TPR2B domain (gray ribbon), based on homology to the TPR1 domain that was co-crystallized with the COOH-terminal EEVD-containing peptide of HSP70 (PDB# 1elw_A), shows that residue 434 would not participate in binding to a putative EEVD ligand (yellow ball-and-stick cluster). In silico mutagenesis of K434R predicts that there would also be no increase of the global folding energy of the TPR2B domain. The seven alpha-helices of typical TPR domain structure are labeled “A1”-”C.” The wild-type and K434R models were submitted to an Atomic Non Local Environment Assessment (ANOLEA) server to compute folding energy; energy differences are in E/*k*T units, where E represents energy; *k*, the Boltzmann constant; and T, absolute temperature. Groups of residues around positions 420 and 434 are predicted to be in a more energetically-favorable conformation in the K434R model.

Stress-induced protein 1 (STI1, OMIM 605063) is a ubiquitously-expressed protein that is involved in coordinating the activities of heat shock proteins (HSPs) in their roles as protein-folding chaperones. The glaucoma-associated variant residue at position 434 is located in helix A3 of the TPR2B domain, which is required to support the individual contacts of the TPR1 and TPR2A domains to HSP70 and HSP90, respectively [26] (see Figure 1 and Figure 2). K434 has been conserved throughout eukaryotic evolution (Figure 1, the homologous position in *S. cerevisiae* is K470) suggesting it plays a crucial role in protein structure or function. A structural homology model of the TPR2B domain (Figure 2, top) was created by alignment with the TPR1 domain co-crystallized with the COOH-terminal peptide of HSP70 which terminates with the EEVD motif [23]. The model demonstrates that either a lysine (K) or arginine (R) amino acid at position 434 does not directly impact the canonical binding cleft that would interact with a putative EEVD-containing ligand. An assessment of the energy required for the mutant and wild-type TPR2B domains to adopt the predicted folds shows that the K434R variant would not require more energy and that it would not affect residues of the potential EEVD-binding pocket (Figure 2, bottom). These analyses suggest that although K434 remained unchanged throughout eukaryotic evolution of STI1, the K434R variant would likely not affect the participation of the TPR2B domain in a canonical TPR-EEVD binding interface, but may subtly alter inter-domain interactions or non-canonical protein–protein interactions involving STI1.

### The effect of the K434R variant on HSP90 function

**Figure 3:**
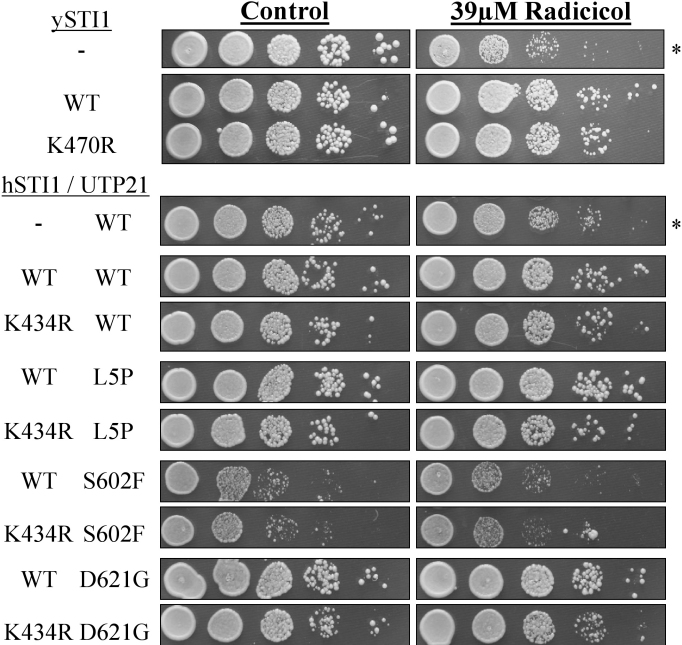
Radicicol sensitivity assay. A null mutation at the yeast *STI1* locus confers sensitivity to the HSP90 inhibitor radicicol (RD) resulting in slow growth at 30 °C on rich media (asterisks). The glaucoma-associated *STI1* variant K434R and the yeast-equivalent K470R do not alter growth on RD plates. Co-expression of yeast *UTP21* mutations (L5P, S602F, D621G) which genetically interact with *sti1*^-^ does not affect sensitivity to RD, either alone or in combination with h*STI1*(K434R).

Haploid yeast with a null *HIS3*-insertion mutation of *STI1* (strain CN11) [20] grow poorly on media containing the HSP90 inhibitor radicicol (RD; Figure 3). We transformed strain CN11 with low-copy number CEN plasmids [34] expressing yeast *STI1* under the control of the natural gene promoter. As shown in Figure 3, growth of the *sti1^-^* null strain is inhibited in the presence of 39 µM radicicol, but normal growth is restored in the strains transformed with either the wild-type or the K470R constructs of yeast *STI1*, indicating that K470R (homologous to the glaucoma-associated K434R variant) did not functionally impair Sti1p's activity in this assay. The K470R variant also did not impair growth on control plates at 30 °C.

We previously developed a haploid yeast strain that carries null chromosomal mutations for both *STI1* and *UTP21* yet express the essential gene *UTP21* from a negatively-selectable plasmid [15]. We co-transformed this strain with recombinant constructs of variant yeast *UTP21* and human *STI1* (as well as wild-type controls), and used negative selection to remove the initial *UTP21* plasmid to create “double-mutants.” To assess what effects these combinations of variants may have on the proper functioning of the HSP90 protein-folding pathway, the strains were grown on plates containing 39 µM RD. Figure 3 demonstrates that the transformed strain lacking h*STI1* but carrying a *UTP21*(WT) plasmid is sensitive to RD and grows slower in its presence. Transformation with wild-type or K434R-bearing h*STI1* plasmids creates strains that are able to grow equally well on control or RD plates. This indicates that K434R has no impact on hSTI1 function in this assay and does not affect strain growth at 30 °C. Further experiments with h*STI1*/*UTP21* double mutants (K434R/L5P, K434R/S602F or K434R/D621G) did not reveal any genetic interactions that compromise the ability of hSTI1(K434R) to rescue RD-sensitivity in this system. Additionally, the double mutants did not show any growth defects at 30 °C.

### The effect of the K434R variant on rRNA processing

**Figure 4:**
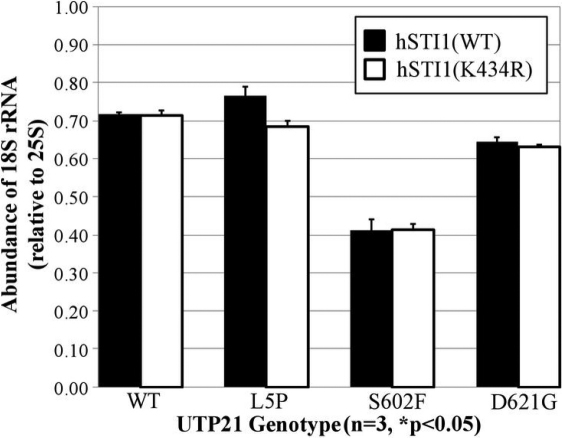
Quantification of rRNA levels. Total RNA from double mutant yeast was analyzed by microchip electrophoresis to quantitate the levels of 18S and 25S rRNA, which are coordinately processed from the same pre-rRNA transcript. When co-expressed with yeast *UTP21* mutations that synthetically interact with the null *sti1^-^* mutation, the K434R variant does not significantly affect 18S rRNA levels.

Synthetic mutations of *UTP21* that are homologous to glaucoma-associated variants of human *WDR36* affect processing of the pre-rRNA transcript in yeast that are deficient for *STI1* [15]. Utp21p is a core protein of the SSU processome that is responsible for the synthesis of 18S rRNA from the unprocessed 35S transcript [16], and a conserved role for human WDR36 has recently been confirmed [18]. 25S rRNA is another end-product of this pathway that is independently derived from the same 35S rRNA transcript. To identify perturbations in the pathway that generates the 18S rRNA product, we quantified the ratio of 18S to 25S rRNA in our double mutant (h*STI1*/*UTP21*) strains using a sensitive microchip electrophoresis platform. Figure 4 demonstrates that in the group of strains transformed with the wild-type h*STI1* construct, expression of the *UTP21* mutations S602F and D621G cause reductions in the relative amounts of 18S rRNA. However, when these *UTP21* mutations were combined with h*STI1*(K434R), there was no further change in relative 18S rRNA levels. Therefore, the K434R variant did not have any measurable effect on UTP21-dependent rRNA processing.

### The effect of the STI1 variants on cell proliferation under heat stress

**Figure 5:**
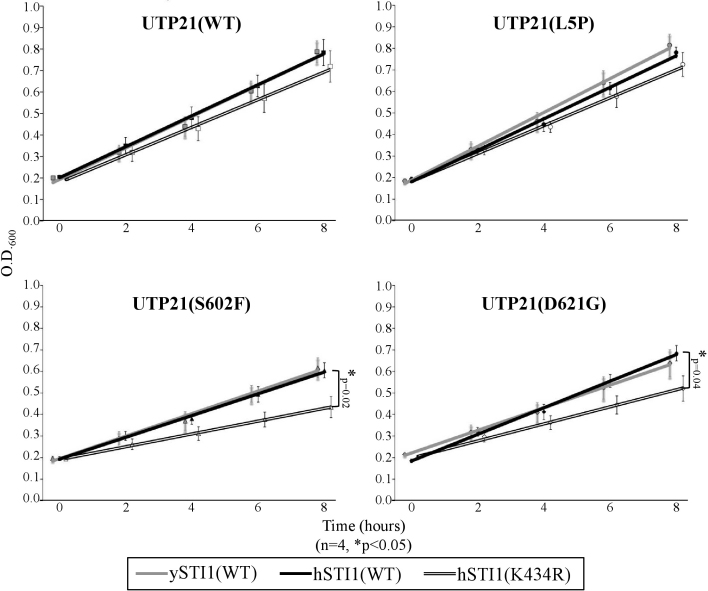
Growth assay of h*STI1*(K434R)/*UTP21* double mutants. Combining specific *sti1^-^*-interacting mutations of yeast *UTP21* with the h*STI1*(K434R) variant decreases the growth rate of liquid cultures at 37 °C. Although expression of the glaucoma-associated K434R variant does not affect growth in the WT or L5P *UTP21* backgrounds, co-expression with the mutations *UTP21*(S602F) or *UTP21*(D621G) significantly decreased the growth rate (asterisk, t-Test of linear slopes, p<0.05, 4 experiments).

To challenge the h*STI1*(K434R)/*UTP21* double mutant strains with an environmental stress, liquid cultures were grown at 37 °C in complete media and their growth curves monitored for 8 h (Figure 5). Culture growth in these conditions followed a linear trend over this time course. K434R/S602F and K434R/D621G strains grew significantly slower than their single mutant counterparts, with doubling times of 7.2 h and 5.6 h, respectively, compared to 4.9 h and 4.3 h for the h*STI1*(WT) counterparts.

**Figure 6:**
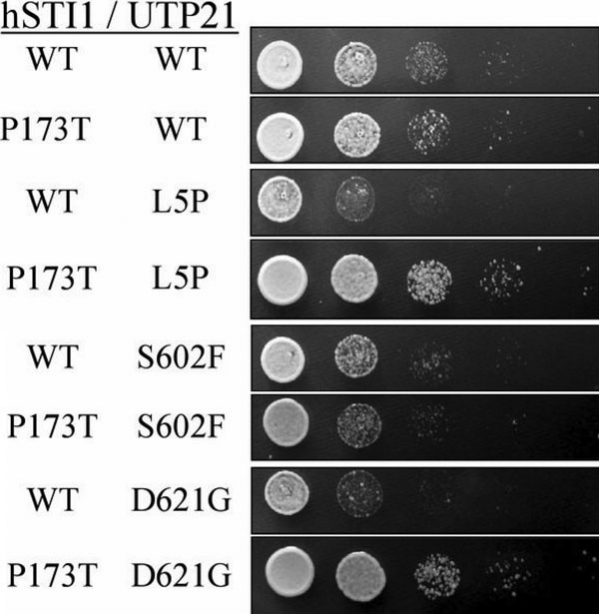
Plate assay of h*STI1*(P173T)/*UTP21* double mutants. The growth of double mutants on complete media at 37 °C was altered in the strains carrying the h*STI1*(P173T) allele. Co-expression of yeast *UTP21* mutations L5P and D621G with h*STI1*(P173T) caused noticeably enhanced growth, while the growth of the S602F double mutant was hindered.

To investigate the generality of the genetic interaction, we tested an additional non-synonymous variant of STI1 identified in dbSNP (rs12789379). The single nucleotide polymorphism that encodes a P173T alteration in humans occurs in a residue that, like K434, has also been conserved in humans and yeast. Although P173 is not located in a domain amenable to homology modeling, it is a component of a sequence motif termed the DP1 domain [24] which appears to contribute marginally to STI1 function [36]. Double mutant strains carrying h*STI1*(P173T) with different alleles of *UTP21* displayed altered growth rates at 37 °C (Figure 6), but not at 30 °C (data not shown), compared to the h*STI1*(WT) counterparts. Curiously, P173T caused faster growth when paired with the L5P and D621G variants of Utp21p but slower growth when combined with S602F.

Taken together, these results show that the combination of adverse environmental conditions and co-expression of separate mutant proteins has noticeable impact on cell proliferation in this model system.

## Discussion

POAG is an ocular disease associated with complex inheritance patterns, multifactorial gene and environment interactions and late age-of-onset. The manifestation and severity of complex diseases can be influenced by a large number of genes each contributing small effects. As well, asymptomatic individuals may actually be undiagnosed carriers of causative mutations who have not yet reached the age-of-onset or experienced environmental insult sufficient enough to trigger disease symptoms. These matters complicate the discovery of the underlying genetic defects of glaucoma. One method to circumvent these problems is the genome-wide association study (GWAS) which concerns genotyping single nucleotide polymorphisms in large patient cohorts. This procedure is being increasingly applied to investigate glaucoma genetics [37-43]. We undertook an alternative approach to gene discovery, by exploring the functional gene network surrounding the POAG-associated gene, *WDR36*.

The contribution of *WDR36* variation to glaucoma pathogenesis is controversial because many of the rare non-synonymous *WDR36* variants sequenced in patients are also present in normal control individuals. This can be explained by the hypothesis that co-segregating heterozygous mutations in two or more unlinked genes are required to develop the disease. Polygenic inheritance of retinitis pigmentosa [44] and of forms of Waardenburg syndrome [45] and Bardet-Biedl syndrome [46] have previously been demonstrated. Supporting the theory that some forms of POAG may also manifest as a polygenic trait, recent work demonstrated that variation in *WDR36* lowers the age-of-onset of the POAG phenotype in members of a large family who also harbor the *MYOC* mutation K423E [47]. Also, we previously established that non-synonymous *WDR36* variants have deleterious effects on cell growth when expressed in the homologous yeast gene, *UTP21*, but that these synthetic effects were dependent on genetic interaction with a null mutation of the gene *STI1* [15].

*STI1* encodes a co-chaperone of the eukaryotic HSP70/HSP90 protein-folding machinery. To investigate whether *STI1* can act as a modifier locus for *WDR36*-associated POAG, we sequenced the coding region of *STI1* in patients heterozygous for *WDR36* non-synonymous variant alleles. One patient was discovered to be doubly-heterozygous, harboring the *WDR36*^L25P^ variation simultaneously with the *STI1*^K434R^ allele. This patient appeared to have an earlier age-of-onset than 10 of the 11 other *WDR36*^L25P^ heterozygotes in our study. However, discovering additional individuals doubly-heterozygous for *WDR36* and *STI1* is necessary to determine the phenotypic consequences of *STI1* variant alleles. Importantly, since our yeast model system revealed that *WDR36* mutations have biologic consequences only when simultaneously present with variation of a second gene (e.g., *STI1*), we hypothesize that glaucoma patients who are *WDR36* heterozygotes also have DNA alterations at other modifying loci and that the presence of the additional alterations is necessary for these individuals to develop the disease. Genes that interact with *WDR36*, such as *STI1*, are excellent candidates for glaucoma modifier loci.

The finding that residue K434 is invariant throughout eukaryotic evolution of STI1 led us to speculate that a mutation at this site could be deleterious to protein function. K434 is situated in the tetratricopeptide repeat domain TPR2B, which is important for the protein's co-chaperone activity in vivo. Although domain TPR2B is structurally similar to both TPR1 and TPR2A and participates in binding to HSP70 [24,26] a bona fide ligand for TPR2B's canonical EEVD-binding pocket has not been conclusively determined by structural analysis. Our homology model suggests that mutation of lysine 434 to arginine would not interfere in the predicted interaction of the TPR fold with a COOH-terminal EEVD peptide, such as that used in TPR1-HSP70 and TPR2A-HSP90 interactions [23]. The model and the charge-conservative nature of this variation also suggest that K434R would not severely disrupt tertiary protein structure. K434R may instead subtly alter the TPR2B domain topology, affecting its inter-domain interactions or its binding with proteins that do not employ the typical TPR-EEVD interface.

We studied expression of the POAG-associated *STI1* variant in haploid yeast expecting to find only subtle phenotypic effects if the variant was indeed contributing to a late-onset polygenic human trait. By making the homologous mutation, K470R, in the conserved site of yeast Sti1p, we discovered that expression of this variant on a null *sti1^-^* background did not affect cell growth at 30 °C either in the presence or absence of the HSP90 inhibitor radicicol. Mutation of the neighboring residue, R469A, which is proposed to be critical for canonical TPR-EEVD binding (as part of the “2-carboxylate clamp”) [23] similarly was reported to have no effect on sensitivity to radicicol [25]. However, expression of R469A did reduce in vivo activity of the glucocorticoid receptor (an HSP70/STI1/HSP90 client protein) [25] suggesting that radicicol-sensitivity does not measure the full extent of HSP90 activity. It follows that the POAG-associated mutation K434R may only subtly affect HSP90 function and in a context-specific manner.

We have demonstrated that the K434R and P173T variants exert their negative effects on cell growth only in the presence of *UTP21* mutations. Therefore Sti1p may have a novel role in Utp21p-dependent processing of the rRNA transcript. It is interesting to note that Utp6p, another SSU processome protein, contains a TPR-like domain which physically interacts with Utp21p [48], suggesting the possibility that Sti1p binds a similar site on Utp21p through one of its TPR domains. Upon examining the levels of 18S rRNA product (relative to levels of the coordinately-processed 25S rRNA product) we found that mutation of *UTP21*can result in quantifiable reductions in the amount of 18S rRNA. However, combinations of *UTP21* mutations with h*STI1*(K434R) did not have any further effect on 18S rRNA levels. The recent report that human STI1 stimulates translation initiation via interaction with PrP^c^ to mediate neuroprotection [30], strongly suggests that co-expressed functional variants of STI1 and WDR36 (which is essential for ribosome biosynthesis) could act additively to impair neurotrophic responses to stress.

The h*STI1*/*UTP21* double mutants were cultured at 37 °C to measure subtle differences in growth rate. Two of the tested *UTP21* mutations, S602F and D621G, displayed synthetic interaction with h*STI1*(K434R). Both of these *UTP21* mutations also previously exhibited synthetic interaction with the null *sti1^-^* mutation (*sti1^-^*/S602F was inviable; *sti1^-^*/D621G showed accumulation of unprocessed rRNA transcript and reduced strain growth) [15]. We tested a K434R/L5P double mutant in an attempt to mimic the genotype of the *WDR36*^+/L25P^;*STI1*^+/K434R^ glaucoma patient discovered in this study. Unfortunately, the NH_2_-terminal regions of WDR36 and Utp21p are not well conserved throughout evolution so the *WDR36*(L25P) variation may not truly be homologous to *UTP21*(L5P). K434R had no effect on growth in the *UTP21*(L5P) background even though *UTP21*(L5P) had the unique ability to partially rescue the temperature-sensitive growth defect of *sti1^-^* [15]. These circumstances, as well as the varied allele-specific effects of the SNP h*STI1*(P173T) in our double mutant strains, indicate there are complex interactions between *STI1* and *UTP21* alleles that are not yet well understood. Further screening in humans will help identify if additional variations of *STI1* are associated with glaucoma and provide further alleles to study for interactions and functional defects in our yeast model system. At present, the low rate of occurrence of the K434R allele (<0.5% of our samples) hinders a statistical analysis of the significance of *STI1* variation to POAG; co-heterozygosity at the two loci examined in this individual may not be the cause of glaucoma in this patient. Additionally, we are only able to assess the effects of genetic variation on conserved physiologic processes at a cellular level in our yeast model; *WDR36*/*UTP21* variants that reside outside of clearly homologous regions may not be suitably modeled. Therefore, the development of an animal model to study co-variation of multiple genes and their effects on the eye will be necessary to gain a proper understanding of the contribution of variation of this gene network to glaucoma phenotypes.

In summary, we found a rare non-synonymous allele of *STI1* in a residue that is invariant throughout eukaryote evolution. This allele, K434R, was found only in a POAG patient doubly-heterozygous for the genes *WDR36* and *STI1*, and was not present in individuals with wild-type *WDR36* (NTG patients and normal controls). This *STI1* variant was expressed in a haploid yeast model system and found to have no effect on *UTP21*'s function in rRNA processing. K434R also did not affect *STI1*'s role in modulating sensitivity to the HSP90-inhibitor radicicol but this does not rule out the possibility that K434R, in a heterozygous state, subtly affects specific, but unknown, protein-folding pathways in the POAG patient. Despite the lack of evidence that K434R perturbs HSP90-mediated protein folding or rRNA processing, K434R was found to confer a growth disadvantage to cells carrying both this allele and specific mutant alleles of yeast's *WDR36*-homolog, *UTP21*.
